# Exploring the why: risk factors for HIV and barriers to sexual and reproductive health service access among adolescents in Nigeria

**DOI:** 10.1186/s12913-022-08551-9

**Published:** 2022-09-23

**Authors:** Morenike O. Folayan, Nadia A. Sam-Agudu, Abigail Harrison

**Affiliations:** 1 Department of Child Dental Health Obafemi Awolowo University, 22005 Ile-Ife, Nigeria; 2 International Research Center of Excellence, Institute of Human Virology Nigeria, Abuja, Nigeria; 3 Institute of Human Virology and Department of Pediatrics, University of Maryland School of Medicine, Baltimore, USA; 4 Department of Paediatrics and Child Health, University of Cape Coast School of Medical Sciences, Cape Coast, Ghana; 5 Department of Behavioral and Social Sciences, Brown University School of Public Health, Brown University, Providence, USA

**Keywords:** Adolescents, HIV, Reproductive health services, Adolescent health services, Rape, Nigeria

## Abstract

**Background:**

Early sexual debut, low educational attainment, history of rape and transactional and intergenerational sex have been associated with HIV infection among Nigerian adolescents, especially females. We sought to understand the “why”, and how to mitigate against these determinants and barriers to addressing adolescent sexual and reproductive health (SRH) and HIV prevention needs.

**Methods:**

This qualitative study generated data from 49 focus group discussions with male and female adolescents living with and without HIV, healthcare workers, members of civil society organizations working with young people, and parents of adolescents living with HIV. Participants were recruited from all six geopolitical zones in Nigeria. Data was analysed with ATLAS.ti software. Hermeneutic units were created, and codes developed from focus group transcripts. Network View Manager was used to create maps of codes, memos and quotations, and relevant quotes were retrieved from transcripts.

**Results:**

Four major themes were identified, relating to individual, parental, community and government roles in reducing the risk of HIV and unplanned pregnancy among adolescents in Nigeria. Individual factors influencing sexual risk behaviours of adolescents include peer pressure, poor risk perception for HIV, and misconceptions about the efficacy of contraceptives and condoms. Respondents entrusted State responsibilities such as facilitation of HIV-affected adolescents’ access to education, rather, to individuals, parents and the community; and placed the blame for rape on rape survivors. Findings also highlighted the inadequacy of health systems to address adolescents’ needs for treatment of sexually transmitted infections and to provide appropriate education on secondary HIV prevention for those living with HIV.

**Conclusion:**

Rigorous studies are needed to understand dynamics between adolescents’ risk behavior, HIV risk perception, parental roles in mitigating HIV risk in adolescents, and the role of communities and government in HIV prevention and treatment for adolescents in Nigeria.

## Introduction

Since the 1994 International Conference on Population and Development Program of Action, several subsequent conferences and multiple guidelines and policies have recognized the importance of prioritizing the sexual and reproductive health (SRH) needs of adolescents [1]. Despite this global commitment, adolescents are still poorly reached, [1, 2] and there are significant challenges in providing SRH services to those who need them [3]. For adolescents, barriers to SRH service access include the stigma associated with teenage pregnancy, cultural taboos about young people's sexuality; inconvenient service hours and facility locations; and high cost of services [4]. Other obstacles include the limited number of adolescent-responsive, confidential health services, legal restrictions, and inadequate parental support for use of these services [5–7]. In addition, adolescents in low- and middle-income countries have inadequate knowledge about sexually transmitted infections (STIs), [8] and only a minority has access to acceptable and affordable STI services [1]. Contraceptive use in these countries is also low, resulting in unwanted pregnancies and pregnancy-related complications that increase morbidity and mortality among adolescent girls [9].

The HIV epidemic is a health crisis that highlights the wide gap in adolescents’ access to SRH services in the African region. Access to HIV testing is also low [5]—only 10% of young men and 15% of young women age 15–24 years in high HIV-burden African countries are aware of their HIV status [10]. This is mainly because of stigma, negative family reactions to HIV testing, low perceived risk among adolescents, poor support of adolescents by healthcare providers, and requirements for parental consent to access health care services [5].

Similar to other high HIV-burden African countries, Nigeria has a poor track record for adolescent HIV testing [5]. Data from Nigeria’s 2017 Multiple Indicator Cluster Survey indicated that 16.7% of adolescents had ever tested for HIV [11]. Early sexual debut increases the risk for multiple sexual partners and for HIV. This risk is mitigated by adequate knowledge of HIV and STIs [12]. However, in the setting of low access to testing, comprehensive knowledge of HIV among adolescents in Nigeria is poor [13]. Nigeria’s demographic health surveys consistently show that the average age at sexual debut among adolescent mothers is approximately 15 years [14]. Despite these reports, SRH programs for adolescents in Nigeria continue to be ineffective [3]; ignoring the multiple individual [15], parental [16–23] community [19], institutional [20-23 and structural [24–26] barriers that make it challenging for adolescents to access needed services. Adolescents living with HIV (ALHIV) in Nigeria are of particular concern, as relatively less attention has been paid to their SRH needs [27]. ALHIV also need access to SRH services to prevent unwanted pregnancies and vertical transmission to HIV-exposed infants [28]. In addition, ALHIV need access to SRH information (such as prevention of secondary transmission of HIV) that enhances their ability to make informed decisions about their sexual health and rights [29]. Our team has highlighted the need to pay attention to these SRH needs to improve long-term HIV outcomes for ALHIV [30, 31].

Our research group has also conducted and published data from our 2014 nationwide study on SRH for adolescents in Nigeria [30, 32–36]. In this study, adolescents were defined as males and females aged 10 to 19 years, in line with Nigerian guidelines [37]. We found that over a quarter (27.3%) of the 1,601 adolescents recruited for the study were sexually active, and only 25% of the 825 adolescents recruited in non-health facility public spaces knew their HIV status [33]. Our findings were similar to those of a 2012 government-commissioned national survey on HIV and reproductive health [38]. Additionally, in our nationwide survey, only 48.4% of sexually active adolescents used modern contraceptives [32]. Condom use at last sexual intercourse was associated with the ability to negotiate its use [32] and good knowledge on HIV transmission and prevention [37]. Early sexual debut [32] and low educational attainment (particularly, lack of tertiary education) [27], history of rape [27, 36], transactional sex [34] and intergenerational sex [33] were risk factors for HIV among female adolescents. History of an STI was a risk factor for HIV infection for male adolescents [27]. We also found that HIV sexual risk behaviors and the use of contraceptives among adolescents in Nigeria differed by gender, HIV status and educational attainment [32]. However, we did not analyze the data to understand the why: factors underlying high risk for HIV and challenges to addressing SRH and HIV prevention services for these adolescents.

This study was therefore conducted to understand determinants of high HIV risk, and barriers to addressing SRH and HIV prevention needs for adolescents living and not living with HIV in Nigeria. This included eliciting reasons why HIV testing was low among adolescents, and identifying factors predisposing to: 1) sexually risk behaviours, 2) HIV infection among females with history of early sexual debut, and 3) educational disadvantages and poor knowledge about modern contraceptives among ALHIV. Finally, we aimed to identify barriers to access and uptake of contraceptive and STI treatment services by adolescents, and how to facilitate improved access.

## Methods

### Study design

This was a qualitative study based on data generated from interviews in our large 2014 nationwide adolescent study. Transcripts of focus group discussions (FGDs) were analyzed to generate results for the specific objectives for the current study.

### Study setting

FGD participants were recruited from six states in Nigeria (one state per each of the six geopolitical zones): Plateau (North-Central), Lagos (South-West), Borno (North-East), Edo (South-South), Imo (South-East) and Kano (North-West). Details of study settings have been previously published [38]. FGDs were conducted at healthcare facilities providing comprehensive HIV services.

### Study population

Study participants were male and female adolescents 10 to 19 years old living with and without HIV, parents/guardians of adolescents living with HIV (ALHIV), health care providers providing services to ALHIV, and members of Civil Society Organisations (CSOs) working with young people living with HIV.

### Standardisation of FGD facilitators

A facilitator and a note-taker were recruited to conduct FGDs in each of the six States. Recruited facilitators were adults (including people living with HIV) experienced in conducting FGDs who resided in the study states and were familiar with local cultures and fluent in local languages. The six facilitators received three days’ training on the study protocol, research ethics and specifics of conducting FGDs on SRH. All facilitators pilot-tested the FGD guides in the field during the training. Modifications were made to FGD guides for the purpose of enhancing cultural sensitivity, improving data collection process, and reducing time spent on data collection. Edits included modifying some words to more appropriate and culturally sensitive terminology, such as changing words like “contraceptives” to local terms representing “family planning”.

### Focus group discussion guides

FGD guides for adolescents and parents were first developed in English (Nigeria’s official language), translated to three major local languages (Yoruba, Igbo and Hausa) and back translated to English by certified translators. Where the need was identified, FGDs were conducted in local languages using the relevant guides. The guides for healthcare providers and CSOs were administered in English. With the exception of the guide for parents of ALHIV, all FGD guides were adapted from the tool used by the Network of Zambian People Living with HIV [39].

The guides for adolescents living with and without HIV had 13 questions on SRH needs, sources of SRH information and services (e.g. condoms, abortion, STI treatment), satisfaction with these services, and challenges and barriers faced in accessing them. Also, participants were asked to discuss their preferred options for obtaining information, counselling and other SRH services.

The guide for healthcare providers had five questions that enquired about the sexual, reproductive and socio-developmental needs of ALHIV, ethical dilemmas encountered during SRH case management, SRH services provided at their health facilities and how accessible these services were to adolescents, challenges their facilities faced in addressing SRH needs of ALHIV; and measures taken to mitigate these challenges. Focus group participants were encouraged to discuss specific cases (while respecting privacy and confidentiality), and to make recommendations for improving ALHIV access to SRH services and other HIV-related health needs in their facilities.

The guide for parents of ALHIV had seven questions, which explored 1) parents’ perceived roles in SRH development of their children; 2) their discussions, and challenges with discussing sex and contraception access and use with their children; 3) their discussions, and challenges with discussing HIV with their children; 4) their perceptions of HIV and SRH needs of their children; and 5) their perception of the quality of HIV and SRH services received at health facilities. Finally, they were asked to make recommendations on how to improve the quality of HIV and SRH services that adolescents need.

The guide for CSOs had four questions that explored perceptions of the SRH needs of ALHIV based on their work experience, and on HIV and SRH services provided to ALHIV by their organisations. The facilitator probed on the availability of family planning services and services for STI and SRH education, prevention of unsafe abortion and post- abortion care, maternal and newborn care, and management of gender-based violence through direct provision or referrals. Participants were also encouraged to share the challenges they faced with providing SRH services to ALHIV, including policy constraints, demand for services, and skills to initiate SRH discussions and identify STI symptoms. The session ended with a discussion on recommendations for improving SRH service access for ALHIV. Under confidentiality and privacy guidelines, discussions were guided by specific cases that had been managed by the respondents.

### Participant recruitment

The target sample size for each focus group was 5 to 10 participants. Solicitation of study participants continued until the maximum size for each FGD was reached.

#### Recruitment of adolescents living with HIV

ALHIV were recruited mainly through their physicians, who introduced the study to parents of minor ALHIV and directly to older ALHIV. Interested parents and adolescents contacted the study team through a study phone number provided by recruiting physicians. Support groups and networks of persons living with HIV in study states were also informed about the study; interested and eligible members would then contact the study team in person or through phone calls.

#### Recruitment of adolescents living without HIV

Adolescents living without HIV and those who did not know their HIV status were recruited from youth centres located in the study states. Interested adolescents were recruited for participation after study information was provided.

#### Recruitment of parents/guardians of adolescents living with HIV

Parents and guardians of ALHIV were recruited similar to ALHIV; prospective study participants were recruited by their clinicians and from among networks and support groups of people living with HIV, at health facilities.

#### Recruitment of health care providers working with young people living with HIV

Healthcare providers contacted for FGDs were identified through the hospital administrator of each health institution after discussions on study eligibility criteria. Nurses, pharmacists and doctors working in HIV treatment centers for six months or longer were contacted individually for study participation after they were provided information.

#### Recruitment of members of CSOs working with young people living with HIV

CSOs working with young people living with HIV in study states were identified through contact with the secretariat of the National Association of Young People Living with HIV. The head of each CSO was contacted and briefed about the study. They were then asked to recommend staff members who had been actively working with young persons for more than six months. The study team then contacted these individuals and sought their interest in participating in the FGD.

### Study procedure and data collection

Eight FGDs were conducted in each State (nine in Borno) as follows: 1) Two FGDs among adolescents 10–14 years old: one for ALHIV and another for adolescents living without HIV or status unknown. Both groups had both male and female participants, with the exception of Borno state, where FGDs were separated by gender in compliance with Islamic guidance; 2) Two FGDs (one each for males and females) were conducted for adolescents age 15 to 19 years living with HIV; 3) One FGD was conducted for male and female adolescents age 15 to 19 years who were living without HIV or untested; 4) Three additional FGDs were conducted: one each among parents/guardians of ALHIV, healthcare providers, and members of CSOs working with ALHIV.

FGDs were conducted in meeting rooms that were conducive for the participants, ensured privacy, and supported quality audio-recording. On arrival, each participant was taken through the informed consent/assent process and given the opportunity to ask clarifying questions. For adolescents 10 to 17 years old, parental consent forms had to be submitted or freshly administered to parents. All participants were provided transport reimbursement (approximately $14 at the time of the study) prior to signing consent forms. Those who wished not to participate were able to leave immediately.

FGD sessions started with facilitators confirming participant approval for audio-recording the session, describing ground rules for conduct, and starting with an ice breaker. FGD guides allowed for probing and exploration of spontaneously generated themes. Notes were taken during each session as a back-up to the audio-recording and to document non-verbal cues. At the end of the FGD, a one-page debriefing form was completed, which summarized basic information about the session (duration, mood of interview, number of participants, facilitators and observers present). A summary report of the discussion was also completed. Audio recordings were transcribed within 72 h, and were deleted from the password-protected computers of all transcribers once transcripts were completed.

### Data analysis

ATLAS.ti software for qualitative data analysis was used to analyse FGD transcripts. Hermeneutic units were created and codes developed from all eight FGD types in order to aggregate the codes that remained after merging. Network View Manager was used to create maps composed of codes, memos and quotations. Relevant quotes were retrieved from transcripts. Themes developed from qualitative analysis were: reasons for low HIV testing rates among adolescents; lower educational attainment among ALHIV compared to adolescents without HIV or unknown HIV status; sexual risk behaviors; early sexual debut and associated increased HIV risk for adolescent females; poor SRH and HIV knowledge; access, uptake, and use of contraceptives among adolescents; the incidence of rape and related risk of HIV infection.

### Ethical considerations

This study was approved by the Nigeria Institute of Medical Research Institutional Review Board and the Health Research Ethics Committees of the State Ministries of Health in Plateau, Lagos, Borno, Edo, Imo, Kano States, and the Federal Capital Territory, Abuja. Written informed consent was obtained for all study participants. Parental consent was obtained for participants 10 to 11 years old; parental permission and participant assent were obtained for adolescents 12 to 17 years old. Participant consent was obtained for adolescents 18 and 19 years old. All staff, researchers, and field workers engaged in this study were trained on research ethics emphasizing the importance of informed consent and confidentiality. No names or personal identifiers were recorded on any study instruments or transcripts.

## Results

A total of 49 FGDs were conducted; eight FGDs per state with one extra FDG done for adolescents age 10 to 14 years living with HIV in Borno who were separated by gender. Duration of the FGDs ranged between 32 and 59 min.

### Reasons for low HIV testing among adolescents

Prior studies have highlighted low HIV testing access and rates among adolescents in high HIV burden African countries [5, 6, 40], and that only 23.7% of adolescents and young persons in Nigeria have ever tested for HIV [16]. We identified that adolescents had low risk perception for HIV: an unknown/untested HIV status was assumed to be indicative of a negative HIV status. The attitude portrayed was: “*I have not tested, I don’t know, and I am likely not to have HIV” (Untested male adolescent-Lagos).* Fear and concerns about how to handle a positive HIV test result were reasons given for adolescents being unwilling to undergo HIV testing. Parents also identified their own non-involvement and avoidance in getting adolescents tested as another reason for low adolescent testing rates.

“I am HIV negative. It is when you know that you have HIV that is when you start feeling the symptoms. It is not that I am afraid to go for test, it’s just that my conscience is clear. I am not a virgin, but it is not only through sex one can contract HIV.” (Untested male adolescent-Plateau).

“I also feel that we should be involved in getting them tested actually, even though we are yet to.” (Parent of ALHIV-Kano)

### Factors predisposing ALHIV to educational disadvantages

Our prior study has highlighted low educational attainment as a risk factor for HIV among adolescents in Nigeria [32]. Females were also worse affected by low educational attainment and were less likely to have tertiary education [41–43]. Focus group discussants noted that many ALHIV were orphans and therefore did not have parents to ensure and support high educational attainment. Most orphan care programs in Nigeria only offer educational support up to secondary school level. The main reason proffered for low educational attainment among ALHIV was financial challenges and lack of family support.

“We also work with orphan and vulnerable children ages between 12–18 yrs. We support them financially to pay their school fees, and materials such as bags, school uniform etc. are given to them. Some of them have been abandoned by their relations, so we look after their welfare” (CSO-Plateau).

### Factors predisposing female adolescents with a history of early sexual debut to HIV

We have previously reported that a significantly larger proportion of female Nigerian adolescents who initiated sex before age 15 years were living with HIV [34]. Rigorous studies also consistently show an association between early sexual debut and HIV among female adolescents, due to genital trauma [44], a history of STIs, having multiple sex partners, inconsistent condom use, transactional sex, a history of sexual violence, and teenage pregnancy [45, 46]. Our previous study could however not establish the direction of the association: were female ALHIV more predisposed to earlier sexual debut, or was early sexual debut the risk factor for HIV among female ALHIV? Focus group discussants shed light on this. Female adolescents in Nigeria had multiple factors predisposing to early sexual debut. Two of these factors were family neglect and peer pressure.

“Yes, they [peers] mock the virgins in their midst and the virgins get uncomfortable and this will force them into sex so as to satisfy their peers.” (CSO-Plateau)

*“Some of the challenges we face are peer pressure. It is difficult for some of us to talk out. Our mates think we don’t have guts, that at this age we should have a romantic relationship.” (Untested/HIV negative adolescent-Borno)*

*“My own advice to parents in my area is that they give birth to many children they cannot cater for and if those children demand for anything, they will say, can’t you go and do what your mates are doing so that you can have money?... and that means having boyfriend. They often push their girls to do these things.” (Untested female adolescent-Plateau)*

Prior studies have discussed biological factors that increase the risk of young females to HIV infection. These include an immature genital tract and cervix that are easily traumatized during sexual intercourse, and increased surface area of the cervix, vagina and possibly the uterus, through which HIV transmission can occur [47]. We explored the perception of respondents on the risk of young girls to HIV infection. None of the respondents highlighted biological factors as a risk factor. Rather, their perceptions about the increased vulnerability of young girls to HIV was socially related: older men seek out younger women for sex.

*“Some people, especially men who are living with HIV will not let their wife know about it. The men will be going to collect drugs secretly. They will not sleep with their wives again; they will rather prefer to sleep with young girls.” (Untested female adolescent-Plateau)*

Delayed sexual debut and at least secondary school educational attainment protect against HIV, especially for adolescent girls [44, 48]. One way of achieving this is through open parental discussion about sex with their adolescent child [49] and promoting girls’ education [48]. However, socio-cultural norms in many Nigerian communities do not support open discussion about sex [6]. Culture and religion- perpetuated rules and norms are significant impediments to the dissemination of sex and sexuality education in schools in Nigeria. Many parents, teachers and other stakeholders have been in opposition to the implementation of educational programs on sex and sexuality in Nigeria [50]. Yet, cultural and religious norms are not altogether opposed to sex and sexuality education, as tutelage of adolescents on manhood and womanhood by kinship systems pre-date modern times [50]. Discussants shared their perspectives on this issue, with emphasis on promoting parent–child communication on sex. Opinions on the content of education on barrier protection and contraception however differed.

*“My daughter is almost 18 years. I know she has a boyfriend. I used to [ tr: usually] discuss about sex and others, and I used to [tr: usually] tell her to abstain”. (Parent of ALHIV-Kano)*

*“I think the more we do campaign on the use of condoms, the more the disease spreads. The best way is to tell them they are not ripe for sex and they should not engage in any sexual relationship. It should be well stressed that engaging in sex is all about risking their dear lives and that abstinence is the key “(CSO-Edo)*

*“To me, it is always good for parents to teach their children about condoms, tell them the advantages and the disadvantages. That is what I will say. The moral aspect, if you are well brought up, you will not just decide that you want to go and have sex. Like my daughter, she is 11 years old, even before she gets to that age, I was lecturing her on HIV and there was one day they shared condoms for us here, I was holding it and I told her if you an adult, this is how to use it, do not start sex and if you do, this is the danger in it.” (Parent of ALHIV-Plateau)*

### Barriers to access and uptake of modern contraceptives by adolescents

The uptake of modern contraceptives among sexually active adolescents in Nigeria is low [51]. In the initial analysis of our 2014 study, adolescents identified that costs and accessibility were not major barriers to accessing contraception [32]. Similarly, adolescents in the current sub-analysis also did not perceive costs as a barrier to contraceptive access. However, poor knowledge about the effectiveness and ease of use of contraceptives; poor societal perceptions about contraception (for example, concerns that young persons who use contraception may have challenges with conception in future); and religious norms that discourage use of contraceptives and promote abstinence, are some reasons that deter adolescents from using contraceptives.

*“For me, I believe in abstinence because in my place, they teach it exclusively to the children. Condoms are not accepted. Just abstain. Someone will want to ask, how? I think this is largely dependent on the parent, if you have parents that are disciplined and have morals, I think they should be able to help these children to abstain. I don’t believe condoms or family planning is the way out” (Parent of ALHIV-Plateau)*

*“I don’t really believe that the condom is safe. I will not use it in the future. I will look for other alternatives, like pills but not sterilization” (Untested male adolescent-Borno)*

*“Family planning is not good because, everything has advantages and disadvantages. The disadvantage is it may lead to spoiling the womb of the girl. The girl may not be able to conceive in the future. The simplest way is that she should use a condom” (Untested female adolescent-Plateau)*

“Condom use is not good” (untested adolescent 10-14yrs-Borno

### Factors contributing to poor knowledge about modern contraceptives among ALHIV

A significantly larger proportion of ALHIV had poor knowledge about modern contraceptives when compared with peers living without HIV [33]. This was a surprising finding, since we expected that ALHIV would have better reproductive health knowledge due to greater contact with healthcare workers. Discussants felt that Nigerian adolescents in general had inadequate information about contraceptives. However, there were no clear insights into why ALHIV have poorer knowledge about modern contraceptives. It was suggested that holding clinics dedicated to ALHIV-responsive care may promote better access to higher quality, needs-oriented SRH information.

*“Yes, I know the withdrawal method, when a man is having sex, he will not release inside, he will release outside. There are also drugs, I don't know the name. There is the one you put under your private part. I don't know the name.” (Untested female adolescent-Plateau)*

*“When we come, they give health talk in the morning before they start seeing patients. The health talk includes kind of food that one can eat even if you are not rich. .... They also distribute condoms and provide good quality water. We used to have mosquito nets but now they are not available.” (Parent of ALHIV-Kano)*

*“They should have their own clinic because they will able to share experience” (Parent of ALHIV-Imo)*

### Rape and risk for HIV infection

Past studies have identified an association between rape and HIV [52, 53]. Our prior work in Nigeria had also confirmed this association [33], plus the high incidence of rape and forced sexual initiation [36], and attendant high-risk sexual behavior adopted as a negative coping mechanism among adolescent rape survivors [54]. Discussants identified that rape was a public menace in Nigeria, with one adolescent identifying rape as the route for her HIV infection.

*“How I got my sickness was through rape, and the man said if I tell my mum or dad he would kill me. He shows me knife and I was afraid. I was just 7 years old then. He was sleeping with me every day and I was fearful to talk to anybody. I bled. My sister saw it but I could not tell her what happened… A test was conducted on the man and on me and we tested positive to HIV.” (ALHIV 15-19 years-Plateau).*

*“There are some of them that are even positive who still face sexual harassment. I heard about two instances [of] demand for sex: the girls will tell them they are positive and those guys will say the girls should be appreciative of their coming to demand sex from them.” (CSO-Plateau).*

*“We have had several cases of rape, involving girls.” (Healthcare provider-Plateau)*

*“Rape has become a serious issue in our country today. I think the female folks need more enlightenment and education concerning this issue of rape. The female folks should be educated on how they move in lonely areas, the way they dress.” (CSO-Edo)*

### Facilitating adolescent access to treatment for sexually transmitted infections

The prevalence of self-reported STIs is very high among adolescents in Nigeria, other African countries, and high-income countries like the US [55–58], andare a risk factor for HIV among male adolescents [56]. In Nigeria, STI treatment is often sought from unqualified persons such as chemists [59] or traditional healers [55]. Unfortunately, in some parts of Nigeria and other African countries, young males are initiated into manhood by through sex with female sex workers [60]. Female sex workers and their sexual partners have high risk for STIs, including for HIV [61]. Respondents felt that limited access to STI management is due mainly to lack of, and misleading information on how to manage genital lesions and abnormal discharge. Additionally, the poor attitude of healthcare workers towards adolescents seeking SRH and STI services prompt the latter to self-medicate or seek treatment outside the hospital.

*“I heard from a Corper* [a graduate from a tertiary health institution on national assignment] *that you can treat gonorrhoea by going to the chemist. I did not have it but visited the chemist to verify. Somebody I met there now said I can use Andrews liver salt and 7Up drink to treat it” (Untested adolescent male-Plateau)*

## Discussion

In our Nigerian study context, adolescents and young adults contribute nearly 35% of new HIV infections [62]; nearly 60% of female adolescents initiate sexual intercourse before 18 years [63], and comprehensive knowledge among adolescents is unacceptably low [64]. Our qualitative study among different actors in adolescent SRH and HIV care explored participants’ insights into reasons underlying determinants of HIV among adolescents, factors affecting adolescent access and uptake of these services, and approaches for mitigation. Many of these factors were barriers occurring at the individual and family level, although key structural barriers at the community and governmental level were also identified. We identified four major themes related to determinants and mitigation of HIV risk and barriers to SRH and STI services among adolescents: 1) individual, 2) parental, 3) community and 4) government roles.

Individual-level factors that influence sexual risk behaviours of adolescents in Nigeria and elsewhere include peer pressure, poor perception of HIV risk, and misconceptions about the efficacy of modern contraceptives [65–67].

Our study identified that parents in Nigeria had a significant role to play in the sexual risk behaviors of adolescents: Poor parental oversight, and parental approval of romantic partners increases sexual risk behaviour of adolescents [68, 69]. On the other hand, parental connectedness reduces sexual risk behavior for both male and female adolescents, and mother–child communication reduces sexual risk behavior for girls [70]. Discussants highlighted how poor parent–child communication has hindered parent–child interactions that could facilitate delayed sexual debut and uptake of contraceptives and HIV testing by sexually-active adolescents. Indeed, available evidence indicates that effective parent–child interactions positively impact adolescent sexual behavior [71–74], including delayed sexual debut [74], better use of contraceptives, and better ability to negotiate safer sex [75]. However, not all parents have the competency to communicate and interact effectively with their adolescents, and not all parents believe adolescents should have access to SRH education.

Schools could address gaps in poor parent–child interactions, but comprehensive sexuality education still rouses opposition from the general public, complicating adoption and implementation of related programs. Despite the social norms that work against school-based delivery of comprehensive sexuality education, Nigeria has managed to make it available to a sizeable proportion of in-school adolescents [76], mainly through the actions of domestic champions [77]. The large out-of-school population in Nigeria limits the impact of school-based curricula: with at least 18.5 million children not in school, Nigeria has the largest population of out-of-school children in the world [78]. Regardless, educational interventions will need to be comprehensive, and address multiple risk factors such as knowledge of HIV prevention, contraceptive safety and effectiveness, and low risk perceptions for HIV and other STIs.

Our study findings reiterate the same concerns and myths on contraceptives from prior surveys and studies in Nigeria, and indicts state agencies for poor application of existing evidence to inform adolescent SRH policies and programs. Studies indicate that adolescents, especially females who bear the burden of using long-term hormonal contraception, have concerns about contraceptive side effects, including weight gain and future fertility [79, 80]. These side effects and misconceptions about them deter adolescents from using contraceptives. As an alternative, adolescents depend on less effective methods, such as withdrawal and traditional remedies [81]. The implication is that adolescents in Nigeria have to contend with short and long-term life changing consequences of poor SRH due to poorly-informed high-risk behavior and lack of access to modern contraception and other evidence-based SRH interventions [82].

We also observed that, across board, respondents entrusted a significant level of what should be state responsibility to individuals, parents and the community. Rape survivors are also blamed for the incidence of rape, with discussants omitting to identify the role of government and the community in preventing rape. However, it is the responsibility of states to develop and implement legal instruments and programs to prevent rape, prosecute rapists, and provide prompt access to medical and psychosocial support for rape survivors [83]. The public needs to be well-informed about the roles and responsibilities of State agencies in addressing the SRH needs of adolescents, and to hold these agencies accountable. Much more has been achieved by domestic champions in this respect since our study was completed, with more States adopting and implementing legal instruments to prosecute rapists [84].

Further state responsibilities include provision of information and education, including comprehensive sexuality education, and addressing Nigeria’s rape epidemic. Access of adolescents—especially female ALHIV—to tertiary education is a challenge whose solution should fall well beyond the remit of individuals and families. Many ALHIV have financial challenges due to orphanhood [85]. Unfortunately, current programmatic support for orphans in Nigeria does not include tertiary education. Other state responsibilities include health systems strengthening to address the needs of adolescents in general for STI treatment, and the needs of ALHIV in particular for education on secondary transmission of HIV. Regulatory barriers to adolescents’ access to SRH services, such as older age of consent, also need to be reviewed and addressed in context.

Our study’s strengths include the large number and diversity of stakeholders that were included, which enabled a wider sampling of relevant insights. Furthermore, data was collected from all six geopolitical zones in Nigeria, which increases generalizability to adolescents living with and without HIV across the country. However, like all qualitative studies, our study is not statistically representative nor population-based, and it is difficult to reflect causality. The dependency on eligible people to refer themselves for study participation may have introduced some selection bias. However, this is not expected to have significantly undermined study findings, as the study population was large enough to capture and reflect diverse perspectives.

## Conclusion

Our findings indicate the importance of evidence-based solutions at individual, family, community and government levels to address HIV risk factors and bridge gaps in the availability of, and access to SRH (including HIV and STI) services and education for adolescents. While generating additional evidence, next steps should involve translating evidence into policy and applying it in implementation. Translation of adolescent SRH evidence into policy in Nigeria should also be cognizant of the differences in needs between adolescents living with, and without HIV, for female and male adolescents, and for those living in societies with uniquely different norms and contexts across the country. Underlying all solutions and their implementation should be empowerment of adolescents to improve their knowledge and agency to reduce personal risks and to optimize their sexual and reproductive health.

## Data Availability

The datasets used and/or analysed during the current study available from the corresponding author on reasonable request*.*

## Abbreviations

ALHIV: Adolescent living with HIV
CSO: Civil Society Organization
FGD: Focus group discussion
HIV: Human Immunodeficiency Virus
SRH: Sexual and Reproductive Health
STI: Sexually transmitted infection
